# Core outcomes in neonatology: development of a core outcome set for neonatal research

**DOI:** 10.1136/archdischild-2019-317501

**Published:** 2019-11-15

**Authors:** James William Harrison Webbe, James M N Duffy, Elsa Afonso, Iyad Al-Muzaffar, Ginny Brunton, Anne Greenough, Nigel J Hall, Marian Knight, Jos M Latour, Caroline Lee-Davey, Neil Marlow, Laura Noakes, Julie Nycyk, Angela Richard-Löndt, Ben Wills-Eve, Neena Modi, Chris Gale

**Affiliations:** 1 Academic Neonatal Medicine, Imperial College London, London, UK; 2 Nuffield Department of Primary Care Health Sciences, University of Oxford, Oxford, Oxfordshire, UK; 3 Neonatal Unit, Rosie Hospital, Cambridge, Cambridgeshire, UK; 4 The Neonatal Unit, Royal Glamorgan Hospital, Llantrisant, Rhondda Cynon Taf, UK; 5 UCL Institute of Education Centre for Longitudinal Studies, London, UK; 6 Department of Women and Children's Health, School of Life Sciences, Faculty of Life Sciences and Medicine, King's College London, London, UK; 7 Paediatric Surgery, Southampton General Hospital, Southampton, UK; 8 National Perinatal Epidemiology Unit, Oxford, UK; 9 School of Nursing and Midwifery, Faculty of Health, Education and Society, Plymouth University, Plymouth, Devon, UK; 10 School of Nursing and Midwifery, Faculty of Health Sciences, Curtin University, Perth, Western Australia, Australia; 11 Bliss, London, UK; 12 Institute for Women's Health, University College London, London, UK; 13 Parent of Neonatal Patient, London, UK; 14 Neonatal Unit, Birmingham City Hospital, Birmingham, UK; 15 Former neonatal patient, London, UK; 16 Neonatal Medicine, Imperial College London, London, UK

**Keywords:** neonatology, outcomes research, evidence based medicine

## Abstract

**Background:**

Neonatal research evaluates many different outcomes using multiple measures. This can prevent synthesis of trial results in meta-analyses, and selected outcomes may not be relevant to former patients, parents and health professionals.

**Objective:**

To define a core outcome set (COS) for research involving infants receiving neonatal care in a high-income setting.

**Design:**

Outcomes reported in neonatal trials and qualitative studies were systematically reviewed. Stakeholders were recruited for a three-round international Delphi survey. A consensus meeting was held to confirm the final COS, based on the survey results.

**Participants:**

Four hundred and fourteen former patients, parents, healthcare professionals and researchers took part in the eDelphi survey; 173 completed all three rounds. Sixteen stakeholders participated in the consensus meeting.

**Results:**

The literature reviews identified 104 outcomes; these were included in round 1. Participants proposed 10 additional outcomes; 114 outcomes were scored in rounds 2 and 3. Round 1 scores showed different stakeholder groups prioritised contrasting outcomes. Twelve outcomes were included in the final COS: survival, sepsis, necrotising enterocolitis, brain injury on imaging, general gross motor ability, general cognitive ability, quality of life, adverse events, visual impairment/blindness, hearing impairment/deafness, retinopathy of prematurity and chronic lung disease/bronchopulmonary dysplasia.

**Conclusions and relevance:**

A COS for clinical trials and other research studies involving infants receiving neonatal care in a high-income setting has been identified. This COS for neonatology will help standardise outcome selection in clinical trials and ensure these are relevant to those most affected by neonatal care.

**What is already known on this topic?**

- Inconsistent reporting of outcomes of limited relevance to former patients, parents and healthcare professionals is an important cause of research waste.
- There is a lack of evidence to guide many neonatal practices, leading to variation in both the care provided and outcomes for patients.
- Core outcome sets (agreed, standardised outcomes to be reported by all trials) have been developed in other fields to improve outcome selection and facilitate meta-analysis.

**What this study adds?**

- Former patients, parents, doctors, nurses and researchers show differences in how they prioritise neonatal care outcomes.
- We have identified 12 outcomes that are important to these stakeholders.
- If these outcomes are reported in a standardised manner by all neonatal research, this will enhance future evidence synthesis.

## Introduction

The neonatal period is crucial to long-term health, and neonatal conditions are the leading cause of disability-adjusted life-year loss.^1^ Preterm birth is a major cause of childhood morbidity^2 3^ and implicated in the pathogenesis of adult non-communicable diseases.^4^ Neonatal care is common; in high-resource settings one in ten babies are admitted to a neonatal unit, a proportion that is increasing.^5^

Unfortunately there is a paucity of high-quality evidence to guide much neonatal practice, leading to variation in clinical care^6 7^ and outcomes.^8 9^ One reason research fails to guide practice is because neonatal meta-analyses rarely provide conclusive recommendations,^10 11^ commonly because trials have used heterogeneous, non-comparable outcomes.^12 13^ A further limitation of neonatal and paediatric research is that the outcomes reported are frequently not meaningful to patients and parents.^14 15^

One solution is the development of a core outcome set: important outcomes identified by key stakeholders using robust consensus methods.^16^ A core outcome set could ensure all future research in a field reports a common subset of clinically meaningful outcomes and reduces research waste by facilitating meta-analysis.^17^ A core outcome set is a minimum set and does not preclude researchers reporting other outcomes where relevant.^16^ The use of core outcome sets for trials is promoted by journals,^18^ Cochrane Review Group editors^19^ and research funders.^20^ Relevant, standardised outcomes are also crucial for observational research,^21 22^ benchmarking,^23^ clinical audit^24^ and quality improvement studies.^25^

## Objective

The objective was to develop a core outcome set for research in neonatology.

## Scope

The core outcome set has been developed to apply to all research involving babies receiving care on any designation of neonatal unit in a high-income setting, with no limitation by gestational age at birth, birth weight or illness severity. It is intended to apply regardless of the specific population of babies, clinical setting or clinical condition that a particular study addresses. The scope was established at the initial steering group meeting following direction from former patients and parents. The parents and former patients all strongly expressed the view that ‘a sick baby is a sick baby’. They were also clear that while it is possible to separate babies on a neonatal unit by gestation, weight or underlying diagnoses, the outcomes that are most important are universal to all these groups. Research involving babies cared for exclusively on labour or postnatal wards or in the community will be excluded as the majority are healthy needing limited medical input.

## Methods

We prospectively registered the study with the Core Outcome Measures in Effectiveness Trials (COMET) initiative (Registration number 842)^26^ and published the study protocol.^27^ Research ethics approval was not required; the project involved consenting adults completing surveys (online supplementary eFigure 1). We formed a steering group to guide the core outcome set development comprising different disciplines, perspectives and expertise (online supplementary eText 1).

10.1136/archdischild-2019-317501.supp1 Supplementary data

We followed COMET initiative methodology^28^ with reference to previous core outcome set development work.^29^ We identified outcomes reported in neonatal trials and qualitative research and then used these to determine a core outcome set using a consensus process (online supplementary eFigure 2).

### Information sources

We undertook a prospectively registered systematic review to identify outcomes reported in neonatal clinical trials.^30^ Randomised controlled trials are widely considered to be the most rigorous method to determine how a treatment affects patients.^31 32^ We searched Cochrane Controlled Trials Register, Cumulative Index to Nursing and Allied Health Literature (CINAHL), EMBASE and Medline from July 2012 to July 2017. Three researchers (SA, SS, JWHW) independently double-screened potentially relevant records based on titles and abstracts and reviewed the full text of selected studies to assess eligibility. Due to the large number of trials identified, only those with over 100 infants in each arm were included. As many trials lead to more than one publication reporting outcomes at different time points, we reviewed all linked publications. Outcomes were extracted and categorised by physiological system.

We undertook a second, prospectively registered^33^ review to identify outcomes from qualitative research.^34^ We searched Applied Social Sciences Index and Abstracts, CINAHL, EMBASE, Medline and PsycINFO from 1997 to 2017. Publications describing qualitative data relating to neonatal care outcomes, reported by former patients, parents or healthcare professionals, were included. Narrative text and grouped outcomes were thematically analysed by physiological system.

The steering group assessed outcomes identified in the two reviews to produce a final inventory in which duplicated or closely related outcomes were grouped. The inventory informed a three-round, online eDelphi survey which was followed by a consensus meeting.

### Participants

We recruited participants for the eDelphi from the following groups:

1. Former patients cared for on a neonatal unit, and parents of neonatal patients, recruited through neonatal charities and social media.
2. Nurses and allied health professionals (including neonatal nurses, midwives, speech and language therapists, occupational therapists, and physiotherapists), recruited through professional journals and associations.
3. Doctors (including neonatologists, obstetricians, paediatric surgeons, general paediatricians, community paediatricians and general practitioners), recruited through the Royal College of Paediatrics and Child Health and professional organisations.
4. Academics and researchers in the neonatal field, recruited through meetings, academic publications and organisations.

Recruitment was international; participants had to have personal experience of neonatal care or research in a high-income setting. We aimed for 30 participants in each group to achieve a total of 120 participants. The sample size followed guidance^35^ and previous core outcome set development.^36^

### Consensus process

**Figure 1:**
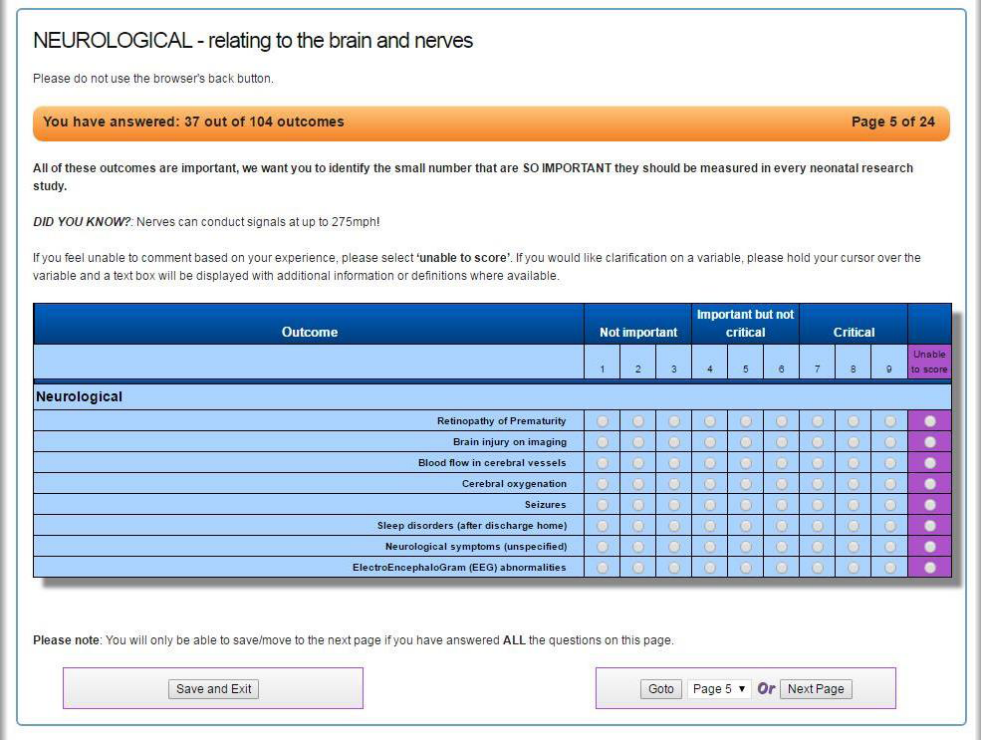
Example screenshot of eDelphi survey.

Participants completed a three-round eDelphi survey^37^ to establish consensus. We ran the eDelphi using DelphiManager software.^38^ To maximise response rates, the survey was kept as short as possible^39^ and extensive demographic data were not collected. In each round we asked participants to rank outcomes between 1 and 9 (with 1–3 meaning ‘limited importance for decision making’ and 7–9 meaning ‘critical for decision making’) following the Grading of Recommendations Assessment, Development and Evaluation guidelines^40^ (figure 1). In round 1, participants could suggest outcomes not identified in the reviews which they felt were important; these outcomes were included in rounds 2 and 3. After each round we collated the results. Before participants reviewed and rescored outcomes in rounds 2 and 3, we presented them with a bar chart showing how each outcome had been scored previously. Each graph combined the scores from all stakeholder groups. We applied predefined consensus criteria to round 3 results.^16^ Provisional core outcomes were those over 70% of participants in each group scored as ‘critical’ and less than 15% of each group scored as ‘limited importance’. Conversely, if over 70% of participants in each group scored an outcome ‘limited importance’ and less than 15% in each group scored it ‘critical’, it was not included. If neither criterion was met, an outcome was classified as ‘no consensus’.

### Consensus between groups

We compared scoring patterns using the first round results to assess agreement between stakeholder groups. The mean scores for each outcome were calculated for each group, and pairwise comparisons were then made between groups. Pearson’s correlation coefficient was calculated for each comparison; differences between coefficients were tested using Fisher’s r-to-z transformation.^41^

### Attrition analysis

We undertook an attrition analysis to ensure the eDelphi results had not been distorted by differences in opinion between those who dropped out and those who completed all surveys. We compared two groups: participants who only took part in round 1 (including those who dropped out during this round) and participants who contributed in all rounds. We compared how these groups scored outcomes in round 1. We used Mann-Whitney U to test for differences in scoring with Bonferroni correction for multiple comparisons (corrected to 5% significance). For outcomes where a difference in scoring was identified, we also tested if the different scoring patterns observed would have changed whether the outcome was considered ‘core’ in round 1 (according to the predefined consensus definition), suggesting attrition affected whether the outcome met the criteria for inclusion in the final core outcome set.

### Consensus meeting

The final prespecified phase was a face-to-face meeting to confirm the final core outcome set based on the eDelphi results. We only invited steering group members and eDelphi participants with additional expertise; the meeting was limited to 16 participants to facilitate discussion.^42^ The consensus meeting remit was limited to refining the final survey results, no new outcomes were considered, and the eDelphi results were paramount. The consensus group were presented the results of the eDelphi and the attrition analysis. They considered whether the identified core outcomes covered all necessary domains, whether there was overlap between outcomes and whether it would be feasible to expect all trials to record each outcome. They discussed the following outcomes in depth: outcomes that met the consensus definition, ‘borderline’ outcomes that narrowly missed the consensus definition (defined as 70% of at least one stakeholder group scored the outcome as ‘critical’) and any outcomes identified during the attrition analysis. Meeting attendees discussed each outcome, then an anonymous vote was held on the question ‘should *the outcome* be included in the core outcome set?’ For inclusion in the final set, 70% of attendees had to vote ‘Yes’. We have published the meeting minutes online.^43^

## Results

This study was completed according to the study protocol.^27^ The only deviation occurred during the review of trials: due to the large number of studies identified, only trials with over 100 neonates in each arm were included. The results of this core outcome set development are reported using COS-STAR (Core Outcome Set–STAndards for Reporting) reporting guidelines.^44^

**Figure 2:**
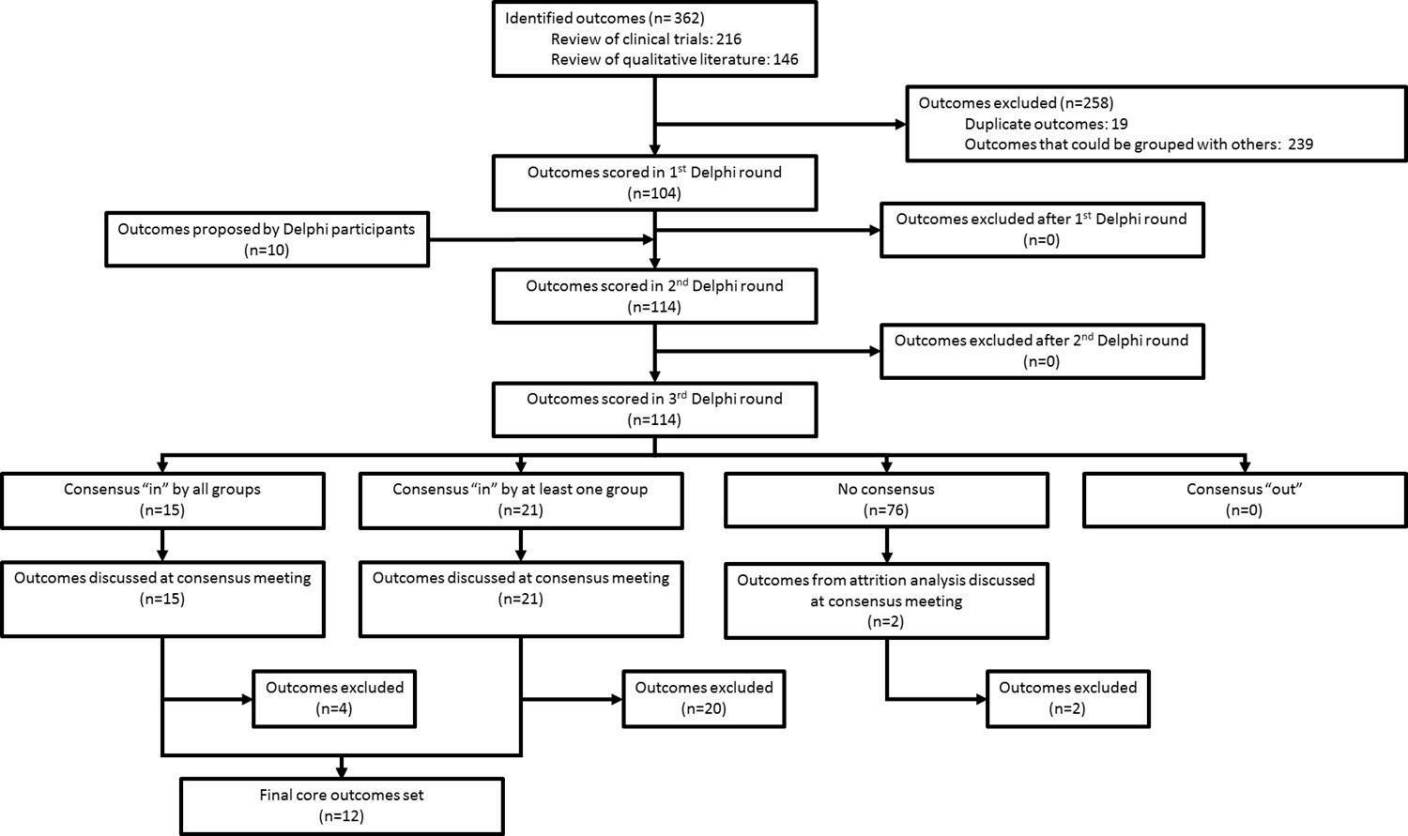
Flow chart of identification and selection of outcomes.

In the review of clinical trials we identified 76 large neonatal trials reporting 216 outcomes, and in the qualitative literature review we identified 62 publications with 146 outcomes.^34^ The steering group reviewed these 362 outcomes, identified 19 duplicates and grouped 239 closely related outcomes. This resulted in a final inventory of 104 outcomes, which were entered into the eDelphi (figure 2) (full list in online supplementary eTable 1). Participants added 10 additional outcomes following the first round (online supplementary eTable 2).

### eDelphi surveys

We recruited a total of 414 participants from 25 countries across 5 continents (online supplementary eFigure 3). The distribution of participants in different stakeholder groups and their participation during the eDelphi are presented in table 1. Participation in all rounds exceeded our target of 120 participants.

**Table 1:** Stakeholder participation across eDelphi rounds

Stakeholder group	Round 1		Round 2		Round 3	
	Started	Completed	Started	Completed	Started	Completed
Parents and patients	244	111	84	61	61	53
Neonatal nurses and allied professionals	53	44	39	38	34	33
Doctors	83	74	71	62	67	59
Neonatal researchers	34	31	29	26	29	28
Total	414	260	223	187	191	173

**Figure 3:**
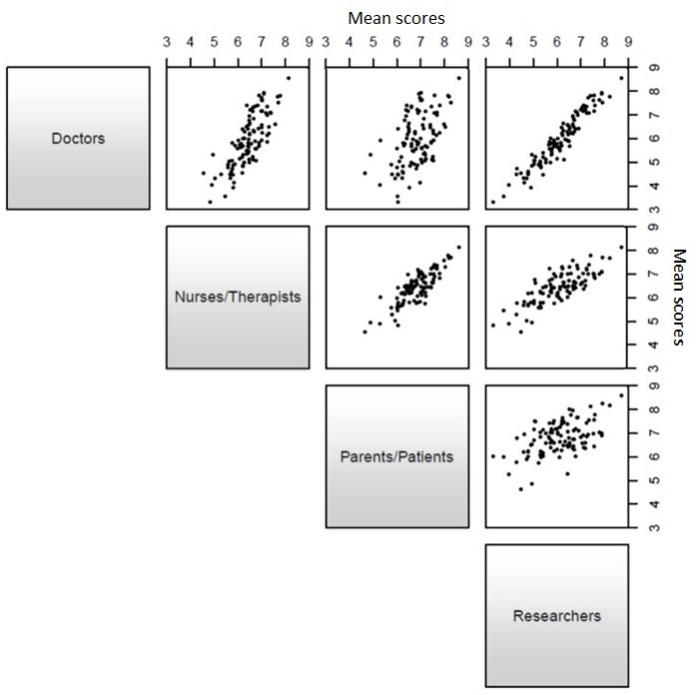
Scatterplots comparing round 1 mean scores (pairwise comparison between stakeholder groups).

Two hundred and sixty participants completed the first round. The mean scores for parents and patients correlated with the scores of nurses and therapists more closely (r=0.83) than with the scores of doctors (r=0.51). The mean scores from doctors correlated most closely with those of researchers (r=0.96). The differences between these correlations were statistically significant (p<0.01). Pairwise comparisons are shown in figure 3.

The final round was completed by 173 participants. The highest scoring outcomes from each stakeholder group are shown in table 2.

**Table 2:** Highest scoring outcomes in round 3 by stakeholder group (outcomes ranked by mean score)

Patients and parents	Nurses and therapists	Doctors	Researchers
Survival	Survival	Survival	Survival
Necrotising enterocolitis	Necrotising enterocolitis	Necrotising enterocolitis	Necrotising enterocolitis
Sepsis	Harm due to treatment*	Sepsis	Sepsis
Brain injury on imaging	Sepsis	Brain injury on imaging	Visual impairment
Harm due to treatment*	Brain injury on imaging	Hearing impairment	Hearing impairment
Parental bonding with baby	Quality of life	Retinopathy of prematurity	General cognitive ability
Pain	Visual impairment	General cognitive ability	Quality of life
Suffering	Pain	Harm due to treatment*	Brain injury on imaging
Parental involvement	Suffering	Ability to walk	Breast feeding
Retinopathy of prematurity	Parental bonding with baby	General gross motor ability	General gross motor ability

*At the consensus meeting ‘Harm from medical treatment’ was redefined as ‘Adverse events’.

The prespecified consensus definition was met for 15 outcomes; these were discussed at the consensus meeting along with 21 outcomes ranked as ‘borderline’. The attrition analysis identified a statistically significant difference between scoring for 19 outcomes (online supplementary eTable 3); for 17 there was no difference in whether the outcome would have been included in the core outcome set. The remaining two outcomes were discussed at the consensus meeting to ensure attrition had not distorted the consensus process.

### Consensus meeting

At the consensus meeting 16 participants representing all stakeholder groups (5 former patients/parents, 3 nurses/therapists, 5 doctors and 3 researchers) discussed and voted on each of the 38 outcomes identified from the eDelphi results. Twelve outcomes were identified for inclusion in the final core outcome set. During discussion the outcome ‘Harm from medical treatment’ was defined as ‘Adverse events’ to allow better alignment with existing classifications of iatrogenic harm. Two outcomes (‘Retinopathy of prematurity’ and ‘Chronic lung disease/bronchopulmonary dysplasia’) relate only to preterm infants and should only be reported by trials involving this group. Meeting minutes and voting results are provided in online supplementary eText 2.

### Core outcome set

The final core outcome set comprises the following:

1. Survival.
2. Sepsis.
3. Necrotising enterocolitis.
4. Brain injury on imaging.
5. Retinopathy of prematurity (*preterm only*).
6. General gross motor ability.
7. General cognitive ability.
8. Quality of life.
9. Adverse events.
10. Visual impairment or blindness.
11. Hearing impairment or deafness.
12. Chronic lung disease/bronchopulmonary dysplasia (*preterm only*).

*(Outcomes were ranked by percentage of round 3 participants who scored each outcome ‘critical for decision making’.)*

## Discussion

Using robust, preregistered consensus methodology, we identified 12 outcomes to be reported in all future trials involving infants receiving care on a neonatal unit in a high-income setting. We hope use of this core outcome set will improve research quality and reduce waste. The core outcome set is a minimum set of outcomes that are so important to all stakeholders that failing to report them will mean that important clinical uncertainties cannot be addressed, both at the level of individual studies and in subsequent meta-analyses.

This core outcome set complements the work by van’t Hooft *et al* 45 in which a core outcome set for interventions to prevent preterm birth was identified. This contained maternal and neonatal outcomes, but the scope was limited to antenatal interventions. A number of core outcome sets have been developed in women’s health^36^; in the newborn period these exist only for gastroschisis^46^ and Hirschsprung’s disease,^47^ with work under way for neonatal abstinence syndrome.^48^ In rheumatology widespread adoption has led to full reporting of the rheumatoid arthritis core outcome set in 80% of relevant trials.^49^ Similar uptake in neonatal research would reduce barriers to meta-analysis^10^ and aid translation of research findings into clinical practice.

A strength of our project was the number of parents and former patients who took part. Our review of trials found no reported involvement of parents or former patients in outcome selection; it is therefore unsurprising that these groups report dissatisfaction with outcomes currently reported in neonatal research.^14^ In our work former patients and parents scored outcomes by importance and could suggest additional important items. Their priorities differed from other stakeholder groups, emphasising the importance of wide involvement in outcome selection.

A limitation of our work was attrition during the eDelphi, which occurred despite efforts to optimise response rates.^39^ The attrition rates in this study are comparable with similar projects.^36^ Explanations for the attrition include the wide range of outcomes (each survey took 20 min) and that participation was voluntary. Former patients and parents were most likely to drop out, perhaps due to their caring commitments.^50^ The attrition analysis identified outcomes where dropout could have skewed scoring patterns and distorted results; those identified were discussed further at the consensus meeting. Participant attrition is common during Delphi surveys; steps to minimise attrition are evolving.^51^ Another limitation is that potential stakeholder groups were not represented (eg, hospital administrators/policy makers). No guidance mandates which groups should be involved in core outcome set development^16^; our project included all groups included in most core outcome set development.^52^

Future work will standardise outcome measures and measurement time points for the outcomes identified. While our review found outcome domains were similar across large neonatal trials, disparate measures and time points meant results were not comparable. Heterogeneity of measures and time points is a known barrier to evidence synthesis.^12^ Defining outcomes like ‘Adverse events’ or ‘Quality of life’, endpoints we have demonstrated to be important to all stakeholder groups, will allow research to report them consistently. Further input from former patients and parents is needed to ensure that outcome measures reflect their lived experiences.^14^ Starting in 2020 we will define measures and time points following OMERACT (Outcome Measures in Rheumatology) 2.0 methodology,^53^ in collaboration with other international efforts.^54 55^ Other core outcome sets have also been developed or are in development in the field of neonatology^46 48^; it is important that overlapping core outcome sets are harmonised to avoid the multitude of incomparable outcomes being replaced by multiple incompatible core outcome sets. The aim is that future research will report the core outcome set alongside trial-specific outcomes; trial-specific outcomes will address a particular research question and core outcomes will provide data for meta-analyses, particularly for prospectively planned meta-analyses.^56^

While core outcome sets are associated with clinical trials, integration with routine data collection will reduce the burden on researchers, facilitate efficient research and improve quality. This will ensure future audit, benchmarking and quality improvement projects are focused on outcomes important to all.

## Conclusion

We have identified a core outcome set for neonatal research. Adoption of this set will standardise outcome selection and ensure these are relevant to those most affected by neonatal care. This will help research translate into improved clinical practice, optimising outcomes for neonatal patients.
