# Supplementary material for: Core outcomes in neonatology: development of a core outcome set for neonatal research
Source: Arch Dis Child Fetal Neonatal Ed. 2019 Nov 15;105(4):425–31. doi: 10.1136/archdischild-2019-317501 (PMC7363790; doi:10.1136/archdischild-2019-317501)
Supplement: Supplementary data [file fetalneonatal-2019-317501supp001.pdf]

Online only supplemental material

eFigure 1: NHS REC decision making tool result
eText 1: Members of COIN Steering Group
eFigure 2: Flowchart illustrating consensus process
eTable 1: List of 104 neonatal outcomes ranked in the Delphi surveys
eTable 2: List of 10 outcomes added at participant suggestion during Delphi Round 1
eFigure 3: Map showing countries where Delphi participants have experienced neonatal care
eTable 3: Results of attrition analysis
eText 2: Minutes of consensus meeting

eFigure 1: NHS REC decision making tool result

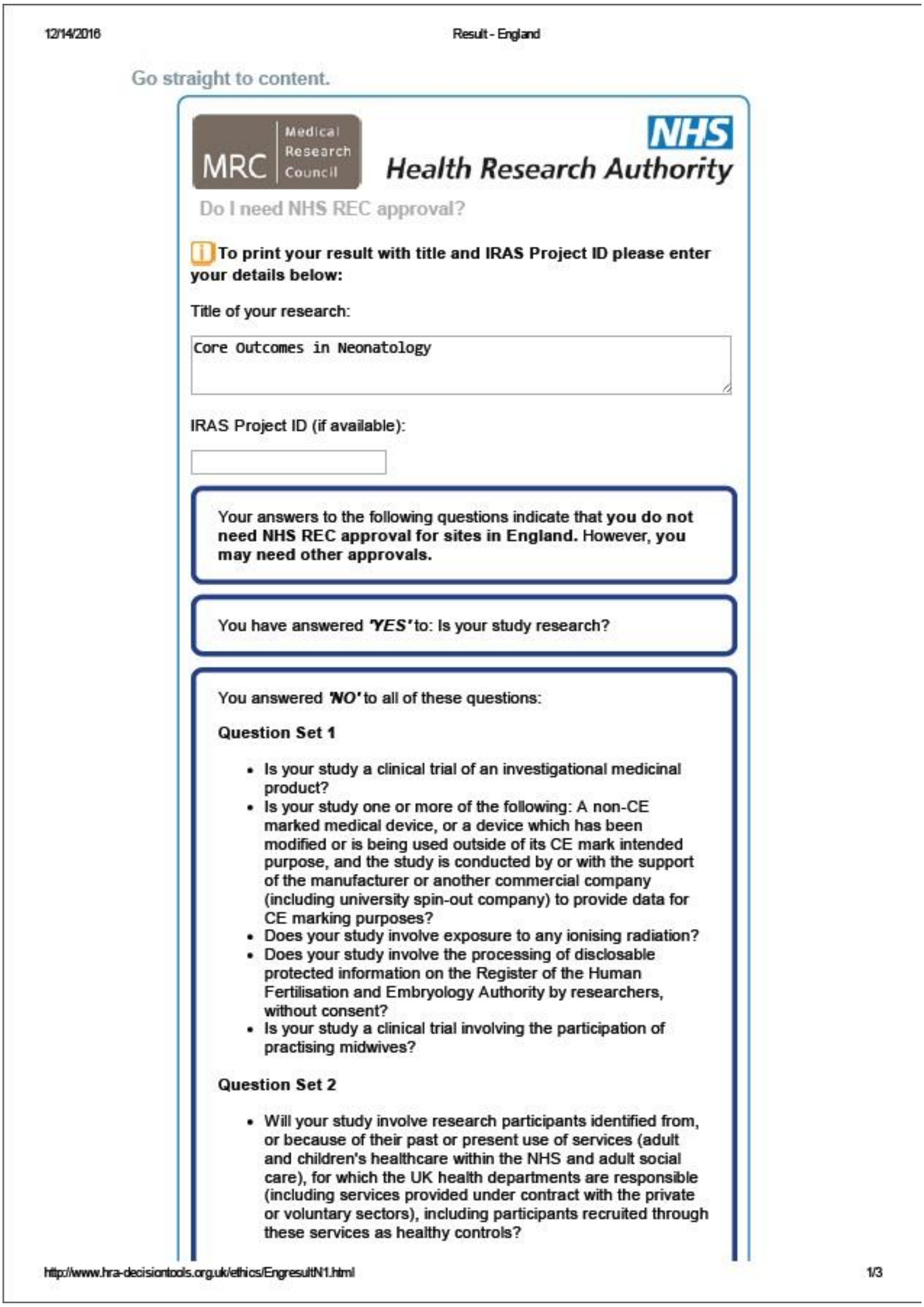

12/14/2016 Result - England

Go straight to content.

MRC Medical Research Council

NHS Health Research Authority

Do I need NHS REC approval?

To print your result with title and IRAS Project ID please enter your details below:

Title of your research:

Core Outcomes in Neonatology

IRAS Project ID (if available):

Your answers to the following questions indicate that **you do not need NHS REC approval for sites in England.** However, **you may need other approvals.**

You have answered ***'YES'*** to: Is your study research?

You answered ***'NO'*** to all of these questions:

Question Set 1

- Is your study a clinical trial of an investigational medicinal product?
- Is your study one or more of the following: A non-CE marked medical device, or a device which has been modified or is being used outside of its CE mark intended purpose, and the study is conducted by or with the support of the manufacturer or another commercial company (including university spin-out company) to provide data for CE marking purposes?
- Does your study involve exposure to any ionising radiation?
- Does your study involve the processing of disclosable protected information on the Register of the Human Fertilisation and Embryology Authority by researchers, without consent?
- Is your study a clinical trial involving the participation of practising midwives?

Question Set 2

- Will your study involve research participants identified from, or because of their past or present use of services (adult and children's healthcare within the NHS and adult social care), for which the UK health departments are responsible (including services provided under contract with the private or voluntary sectors), including participants recruited through these services as healthy controls?

http://www.hra-decisiontools.org.uk/ethics/EngresultN1.html 1/3

- Will your research involve collection of tissue or information from any users of these services (adult and children's healthcare within the NHS and adult social care)? This may include users who have died within the last 100 years.
- Will your research involve the use of previously collected tissue or information from which the research team could identify individual past or present users of these services (adult and children's healthcare within the NHS and adult social care), either directly from that tissue or information, or from its combination with other tissue or information likely to come into their possession?
- Will your research involve research participants identified because of their status as relatives or carers of past or present users of these services (adult and children's healthcare within the NHS and adult social care)?

Question Set 3

- Will your research involve the storage of relevant material from the living or deceased on premises in the UK, but not Scotland, without an appropriate licence from the Human Tissue Authority (HTA)? This includes storage of imported material.
- Will your research involve storage or use of relevant material from the living, collected on or after 1st September 2006, and the research is not within the terms of consent from the donors, and the research does not come under another NHS REC approval?
- Will your research involve the analysis of DNA from bodily material, collected on or after 1st September 2006, and this analysis is not within the terms of consent for research from the donor?

Question Set 4

- Will your research involve at any stage intrusive procedures with adults who lack capacity to consent for themselves, including participants retained in study following the loss of capacity?
- Is your research health-related and involving prisoners?
- Does your research involve xenotransplantation?
- Is your research a social care project funded by the Department of Health?

If your research extends beyond **England** find out if you need NHS REC approval by selecting the *'OTHER UK COUNTRIES'* button below.

OTHER UK COUNTRIES

If, after visiting all relevant UK countries, this decision tool suggests that you do not require NHS REC approval follow this link for final confirmation and further information.

Print This Page

NOTE: If using Internet Explorer please use browser print function.

About this tool **Feedback** **Contact** **Glossary**

eText 1: Members of COIN Steering Group

Steering Group Chair

Marian Knight

Professor of Maternal and Child Population Health. National Perinatal Epidemiology Unit, Oxford

Project Management Team

Chris Gale

Clinical Senior Lecturer in Neonatal Medicine. Imperial College, London

James Webbe

Clinical Research Fellow. Imperial College, London.

Steering Group

Elsa Afonso

Neonatal Staff Nurse. Cambridge University Hospitals NHS Foundation Trust, Cambridge.

Iyad Al-Muzaffar

Consultant Neonatologist and Parent of preterm baby. Cwm Taf University Health Board, Wales.

Ginny Brunton

Midwife and qualitative methodologist. UCL Institute of Education, London.

James Duffy

Doctoral Research Fellow in Obstetrics and Gynaecology. Nuffield Department of Primary Health Sciences, Oxford.

Anne Greenough

Professor of Neonatology and Clinical Respiratory Physiology. King's College, London.

Nigel Hall

Associate Professor of Paediatric Surgery, University of Southampton, Southampton

Jos Latour

Professor in Clinical Nursing. University of Plymouth, Plymouth.

Neil Marlow

Professor of Neonatal Medicine. University College, London.

Neena Modi

Professor of Neonatal Medicine. Imperial College, London.

Laura Noakes

Parent of neonatal patient

Julie Nycyk

Consultant Neonatologist, Sandwell and West Birmingham Hospitals NHS Trust, Birmingham.

Mehali Patel

Research Engagement Officer. Bliss, London.

Angela Richard-Londt

Parent of neonatal patients

Ben Wills-Eve

Ex-neonatal patient

eFigure 2: Flowchart illustrating consensus process

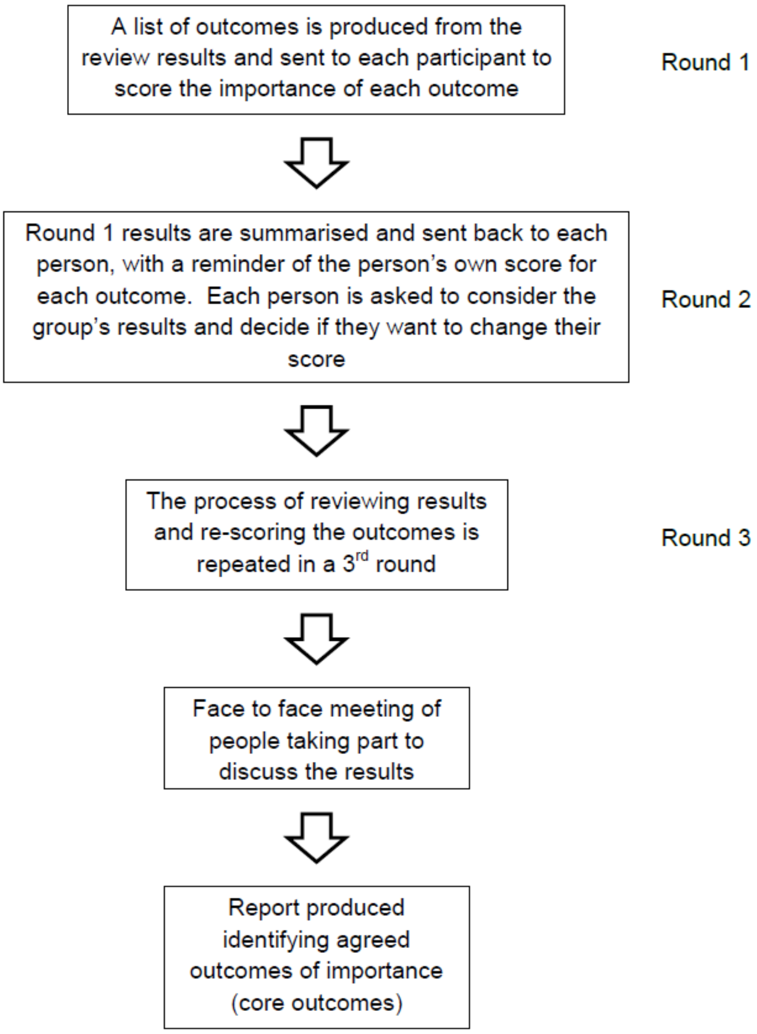

eTable 1: List of 104 neonatal outcomes ranked in the Delphi surveys

Outcome Domain	Outcome
Survival	Survival
Respiratory	Mechanical ventilation
	Chronic Lung Disease (CLD) or Bronchopulmonary Dysplasia (BPD)
	Oxygen at discharge home
	Oxygen saturations
	Oxygen requirement
	Use of non-invasive respiratory support
	Use of surfactant
	Complications of Respiratory Support
	Respiratory Distress Syndrome (RDS)
	Effort of breathing
	ExtraCorporeal Membranous Oxygenation (ECMO)
	Inhaled Nitric Oxide
	Childhood asthma (after discharge home)
	Excessive secretions
	Frequent respiratory illnesses (after discharge home)
Cardiovascular	Patent ductus arteriosus (PDA)
	Measures of blood flow
	High blood pressure and management
	Low blood pressure and management
	Damage from inadequate blood supply
	Echocardiographic measures of heart function
	Circulatory collapse
Gastrointestinal	Necrotising enterocolitis
	Enteral feeding
	Parenteral Nutrition (PN)
	Blood flow to intestines
	Spontaneous intestinal damage
	Feeding intolerance
	Gastrointestinal bleeding
	Breastfeeding
	Gastro-oesophageal reflux disease (GORD)
	Daily nutrition intake
	Frequency of defaecation
	Type of milk
	Oral feeding after discharge home
	Structural gastrointestinal abnormality
Neurological	Retinopathy of prematurity
	Brain injury on imaging
	Indirect measures of blood flow in cerebral vessels
	Indirect measures of oxygen delivery to the brain

**Outcome Domain**	**Outcome**
Neurological	Seizures
	Sleep disorders (after discharge home)
	Other neurological problems
	ElectroEncephaloGram (EEG) abnormalities
Genitourinary	Poor kidney function
Infection	Sepsis
	Colonisation
	Antimicrobial use
	Susceptibility to infection (after discharge)
	Pneumonia
Skin	Skin integrity
	Skin injuries
	Appearance of scars
Surgical	Need for surgical operations
	Need for a stoma
	Meconium passage
Development – Gross Motor	General gross motor ability (walking/sitting etc.)
	Ability to walk
	Need for physical therapy (after discharge home)
	Ability to undertake sport
Development – Fine Motor	General fine motor ability (writing/dressing etc.)
	Ability to feed themselves
Development - Cognitive	General cognitive ability
	Need for educational support
Development – Special Senses	Visual impairment or blindness
	Hearing impairment or deafness
Development – Speech and Social	General communication ability
	Social difficulties (after discharge home)
	Speech delay
Psychosocial	Psychiatric disorder (after discharge home)
	Autism after discharge home
	Childhood happiness after discharge
	Family and peer relationships
Healthcare Utilisation	Readmission in childhood
	Duration of neonatal stay
	Frequent appointments and treatments
	Healthcare costs
Outcomes Related to Parents	Parental involvement
	Parental ill health
	Support for parents
	Parental competence
	Parental ability to work
	Parental bonding with their baby

**Outcome Domain**	**Outcome**
Outcomes Related to Healthcare Workers	Effective communication
	Effective caring relationships with parents
	Team-working by healthcare professionals
	Harm due to medical treatment
General Outcomes	Normality after discharge
	Suffering
	Pain
	Growth
Miscellaneous	Stability of vital signs
	Haematological blood test results
	Biochemical blood test results
	Infant body temperature
	Fluid intake
	Apgar scores
	Self identifying as premature
	Vitality
	Treatment of jaundice
	Liver failure
	Resuscitation at birth
	Treatment with medications

eTable 2: List of 10 outcomes added at participant suggestion during Delphi Round 1

Outcome Domain	Outcome
Genitourinary	Continence in later childhood
Surgical	Tracheostomy
Healthcare utilisation	Invasive procedures
	Need for transfer
Outcomes Related to Parents	Parental satisfaction with care
	Parental psychological harm resulting from NICU experience
Outcomes Related to Healthcare Workers	Communication with former neonatal patients
	Staff satisfaction with care provided
General Outcomes	Quality of life
	Ability to live independently

eFigure 3: Map showing countries where eDelphi participants have experienced neonatal care

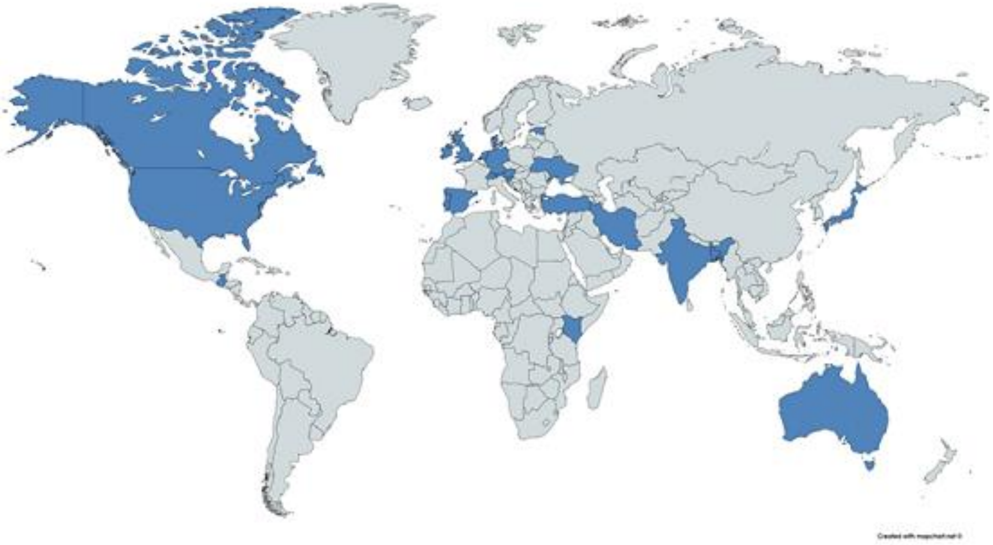

Countries represented:

Australia
Austria
Bangladesh
Canada
Denmark
Estonia
Germany
Guatemala
India
Iran
Ireland
Japan
Kenya
Netherlands
New Zealand
Portugal
Slovenia
Spain
Sweden
Switzerland
Turkey
Ukraine
United Kingdom
United States of America

eTable 3: Results of Attrition Analysis

Outcome	Round 1 only participants Mean score	All Rounds participants Mean score	Round 1 only participants % scoring 7-9	All Rounds participants % scoring 7-9
Frequency of defaecation	6.05	4.21	33	12
Susceptibility to infection (after discharge)	7.21	5.65	64	29
Echocardiographic measures of heart function	6.82	5.45	47	24
Excessive secretions	5.79	4.39	32	11
Oxygen saturations	6.95	5.76	55	32
Effort of breathing	6.52	5.31	47	23
Gastro-oesophageal reflux disease (GORD)	6.38	5.04	38	17
Colonisation	6.63	4.91	41	16
Indirect measures of blood flow in cerebral vessels	7.37	5.97	59	35
Indirect measures of oxygen delivery to the brain	7.38	5.93	57	35
Pneumonia	7.1	5.93	55	33
Feeding intolerance	6.88	5.9	56	33
Support for parents	7.42	6.33	70	39
Respiratory Distress Syndrome	7.1	6.19	56	42
Frequent respiratory illnesses (after discharge home)	6.86	6.09	55	35
Effective caring relationships with parents	7.4	6.44	72	44
Team-working by healthcare professionals	7.43	6.45	78	46
Treatment of jaundice	6.07	4.83	35	15
Self -identifying as premature	5.71	4.37	37	9

Detailed results of the attrition analysis. Comparison of scoring patterns between participants who dropped out after round 1 (‘Round 1 only’ group) and those who completed all rounds (‘All Rounds’ group). All scores are from round 1. Outcomes are ordered by significance of difference seen (from most to least significant, all differences significant at p<0.05 with Bonferonni correction for 104 comparisons).

eText 2: Minutes of COIN consensus meeting

COIN Consensus Group Meeting

12th September 2018: Structured Minutes

Meeting Chair: Prof Marian Knight

Present: Iyad Al-Muzaffar, Helen Chitty, Jennifer Deeney, Chris Gale, Nigel Hall, Juliette Lee, Caroline Lee-Davey, Claire Marcroft, Neil Marlow, Geoff Miller, Julie Nycyk, Angela Richard-Londt, James Webbe, Ben Wills-Eve.

Observer: Rachael Wood

Apologies: Elsa Afonso, Ginny Brunton, James Duffy, Anne Greenough, Jos Latour, Neena Modi, Laura Noakes, Martin Ward-Platt.

I. Welcome and Introduction Presentation

Welcome extended to all present by JW. Background to core outcomes sets presented by JW along with evidence of problems caused by poor outcome selection in neonatal research. COIN project plan recapped (outcomes identified by systematic reviews, three round Delphi process used to score outcomes by importance before final consensus meeting to identify core outcomes set). Aims for day discussed: identify core outcomes set that is pragmatic and realistic.

Feedback from peer review of presentations/publications discussed. Recurrent issue that not all outcomes will be relevant to babies of different gestational ages. Suggestion made by JW that some outcomes only applicable to certain ages. Suggestion by NM that outcomes only relevant to preterm infants be identified separately. Suggestion unanimously approved by all present.

II. COIN Delphi Process

Results of COIN Delphi work presented by JW. Following points discussed:

- Recruitment exceeded planned numbers in all groups. Over 30 participants in each stakeholder group throughout process (except neonatal researchers, where 28 completed all rounds). Attrition noted, especially among parent group.
- Improvement in consensus between groups over three rounds noted.
- Results of Round 3 noted with 15 outcomes meeting predefined consensus criteria (all four stakeholder groups having >70% scores 7-9): Survival, Sepsis, Necrotising enterocolitis (NEC), Brain injury on imaging, Retinopathy of prematurity, General gross motor ability, General cognitive ability, Visual impairment or blindness, Pain, Quality of life, Hearing impairment or deafness, Seizures, Harm due to medical treatment, Need for surgical operations, and Ability to walk.
- Further group of borderline outcomes noted where at least one stakeholder group had >70% score 7-9: Chronic lung disease/Bronchopulmonary dysplasia, Suffering, Parental bonding with their baby, Resuscitation at birth, Growth, General fine motor ability, Normality after discharge, General communication ability, Effective communication, Parental involvement, Oxygen at discharge home, Ability to feed themselves, Ability to live independently, Mechanical Ventilation, Tracheostomy, Damage due to inadequate blood supply, Need for educational support, Circulatory collapse, Breastfeeding, Complications of Respiratory Support, and Other neurological problems.
- Attrition analysis presented, comparison of scoring in Round 1 between those who participated in only Round 1 and those who completed all 3 rounds. Only two outcomes identified that showed statistically significant difference in scoring with difference in whether it would be included in final core outcomes set. These outcomes to be reviewed during meeting: Effective caring relationship with parents, team-working by healthcare professionals

These outcomes would be discussed individually to determine which would be included in the core outcomes set.

III. Discussion of outcomes

It was agreed by all that minutes for this section would not name individuals, but contributions would be listed by stakeholder group. For each outcome Delphi results reviewed followed by period of discussion. After

discussion vote held with all voting In or Out (except MK abstaining as chair and RW abstaining as observer). If >70% of votes are In outcome to be included in core outcomes set. Any other result means outcome not in core outcomes set.

For each outcome the major discussion points have been summarised by stakeholder group with the results of the vote.

PROVISIONAL CORE OUTCOMES SET

Survival

Delphi results reviewed. All stakeholders agreed that outcome of paramount importance

Vote: In 100%

Out 0%

Result: Included in core outcome set

Sepsis

Delphi results reviewed.

Former patients and parents emphasised importance of this outcome. Interweaving of sepsis with many other outcomes noted.

Nurses and therapists discussed how sepsis affects all aspects of clinical practice.

Doctors questioned whether sepsis was an important outcome in all trials, but recognised that it is a crucial part of the broad clinical picture and can have unexpected consequences.

Agreed by all that this is measurable, and there is no significant overlap with other outcomes.

Vote: In 100%

Out 0%

Result: Included in core outcome set

Necrotising enterocolitis

Delphi results reviewed.

Former patients and parents discussed that this was an outcome that all parents know about, and that for many it was their biggest fear. Impact it has on feeding practices noted. Range of severity of illness discussed.

Researchers mentioned that this can be triggered by all sorts of interventions. It does occur in term infants (although it is rare).

Agreed by all that this is measurable, and there is no significant overlap with other outcomes.

Vote: In 100%

Out 0%

Result: Included in core outcome set

Brain injury on imaging

Delphi results reviewed.

Former patients and parents mentioned that brain injury on scans was important, but not always predictive of long term outcome.

Nurses and therapists reported that brain injury was important particularly due to the relation with development effects.

Doctors felt that this outcome was very important, but raised the issue that a range of imaging modalities are available and that not all babies need imaging. Questions about whether the injury on imaging is important or the consequences of that injury.

All groups agreed that this outcome relates to a brain injury occurring during the initial neonatal admission detected on imaging (of any modality).

Vote: In 100%

Out 0%

Result: Included in core outcome set

Retinopathy of prematurity

Delphi results reviewed.

Former patients and parents noted that this outcome only relates to some preterm infants. They discussed that if it was missed the effects could be far-reaching.

Doctors mentioned that only certain infants will be screened.

Researchers clarified that in the UK it is only screened for in infants with birthweight under 1250g or born before 31 weeks gestational age. They discussed how it can be affected by the quality of the overall care received by babies, although if treated correctly it should have few long term consequences.

It was recognised by all that screening varies by location. Inclusion in the core outcome set would not require any change in practices, it would simply require that the results of this screening are reported in a standardised manner.

Vote: In 100%

Out 0%

Result: Included in core outcome set

General gross motor ability (walking/sitting etc.)

Delphi results reviewed. All stakeholders agreed unanimously that this related to gross motor ability measured after the neonatal period.

Former patients and parents discussed that most preterm infants will be ‘delayed’, but many will catch up. They stated clearly that it was not simply due to brain injury, but could be related to other factors.

Nurses and therapists talked about the wide variations seen even in normal development which can be even wider in premature infants. They talked about how difficult it can be to predict later effects at an early age.

It was discussed that this outcome does have significant overlap with the outcome “ability to walk”.

Vote: In 100%

Out 0%

Result: Included in core outcome set

Ability to walk

Delphi results reviewed.

Nurses and therapists talked about how this is an outcome parents often asked about. They discussed how it can be very variable.

Doctors confirmed the significant overlap with "general gross motor ability". Including it in the core outcome set would be a major duplication. They suggested that this is a single, very variable outcome.

It was discussed that this outcome does have significant overlap with the outcome "general gross motor ability". All agreed that this outcome should be considered as a possible measure of gross motor ability (especially as it will be necessary to think pragmatically about what can be measured routinely).

Vote: In 0%

Out 100%

Result: Not included in core outcome set

General cognitive ability

Delphi results reviewed. All stakeholders agreed that this was a measurable outcome.

Former patients and parents discussed this could be a major cause of ongoing problems. They discussed how the realisation that problems exist may not occur until the age of 12 to 13 and that problems can then extend into the teenage years and beyond. They strongly emphasised that they would want this to be measured over as long a time period as possible due to the time gap that can exist between birth and the onset of problems.

Researchers discussed how issues could be multifactorial. They raised the issue that including this in the core outcome set could help raise awareness of the importance of long-term outcomes with researchers, funders and other groups.

Vote: In 100%

Out 0%

Result: Included in core outcome set

Quality of life

Delphi results reviewed. All stakeholders agreed that this can be measured (commonly done using questionnaires).

Former patients and parents stated clearly that this was not just about health. They reported how it was related to many factors. They discussed how many former preterm infants were very aware of "what might have been". This can mean that the impact of disabilities on their quality of life is reduced. They talked about how they felt it was a very important outcome, with relevance beyond clinical trials. Social interaction was identified as a major component affecting quality of life. They stated clearly that quality of life is highly individual and should not be specified by an external source.

Nurses and therapists felt that the timing of this outcome would be crucial and would have implications for how it was measured and how relevant it was to patients' lives.

Doctors agreed that this outcome is important. They were concerned that measuring this in all trials might not be feasible.

Researchers were concerned that this was a very broad outcome which would be difficult to measure with a single measure. They talked about the WHO definition of quality of life (which relates to the ability of individuals to integrate and participate in society). They recognised that this outcome did encompass many important aspects of quality/function.

All stakeholders agreed that identifying an appropriate, robust outcome measure would be crucial if this was to be included in the core outcome set.

Vote: In 57%

Out 43%

Result: Not included in core outcome set

Harm from medical treatment

Delphi results reviewed.

Former patients and parents talked about how this is not just avoidable harm, treatment often involves a balance of risks and benefits (for example prolonged use of PN may lead to liver disorders but the benefits still outweigh the harms of treatment). They were uncertain that a single compound measure would be useful if some ‘harm’ was acceptable while some was potentially avoidable.

Nurses and therapists felt that ‘adverse events’ should be reported in interventional trials. They were concerned that this would be difficult to pre-define.

Doctors identified that it can be difficult to define ‘harm’. All treatments have side-effects and causality is rarely clear cut. They felt that there was significant overlap with the concept of adverse events. It was identified that this does cover one of the OMERACT (Outcome Measures in Rheumatology) framework (Boers et al., 2014) domains.

Researchers discussed that this was vague. They identified that it could be difficult to prove causality. They stated strongly that adverse events should be reported, but are not universally discussed. One problem with limiting such an outcome to adverse events attributed to the intervention is that it is often difficult to be certain of attribution and hence important adverse events may be hidden.

All agreed that this outcome would need a precise definition. All agreed unanimously that this outcome would be best titled as “adverse events”. When the definition is identified this will need to consider both all adverse events and adverse events specifically attributed to the treatment.

Vote: In 93%

Out 7%

Result: Included in core outcome set (as “adverse events”).

Visual impairment or blindness

Delphi results reviewed. All stakeholders agreed that this was a measurable outcome.

Former patients and parents agreed that this is important. They mentioned that other sensory domains are also important.

Nurses and therapists, doctors and researchers emphasised that there are many other causes beyond retinopathy of prematurity and so this does not duplicate the outcome ‘retinopathy of prematurity’.

Vote: In 100%

Out 0%

Result: Included in core outcome set

Hearing impairment or deafness

Delphi results reviewed. All stakeholders agreed that this was a measurable outcome.

Former patients and parents agreed that this is important. They felt that it was important to measure this at a later stage as early measures may not be totally predictive of later hearing impairment.

All stakeholders agreed that this was an important outcome.

Vote: In 100%

Out 0%

Result: Included in core outcome set

Need for surgical operations

Delphi results reviewed. All stakeholders agreed that this was a measurable outcome.

Former patients and parents discussed that there was a major difference between needing an operation and having one. They stated that if surgery occurred it was assumed that it was needed. They were unclear whether having an operation was important or whether the results of the surgery were what really mattered.

Doctors highlighted the range of operations (commonly related to gastrointestinal issues, patent ductus arteriosus ligation and treatment for retinopathy of prematurity). They mentioned that the context of surgery could be very different and that not all operations were the same. Corrective surgery for a congenital cardiac disorder could be essential for ongoing life and be unavoidable. In contrast an emergency laparotomy for NEC might be an adverse consequence of other aspects of care.

All stakeholders agreed that surgical operations should be reported as adverse events. Significant overlap with this domain agreed by all.

Vote: In 0%

Out 100%

Result: Not included in core outcome set

Pain

Delphi results reviewed. All stakeholders agreed that adequate pain management during trials is critical and must be given the utmost priority (regardless of whether pain is considered a core outcome).

Former patients and parents were split over the importance of this outcome. Some felt that it was “very key” and could be measured throughout life, with pain scales already in use and new methods of measuring pain emerging with the latest research. Others felt that the long-term adverse effects of neonatal pain were minimal.

Nurses and therapists felt that assessing neonatal pain could be subjective, but they did feel that carers could tell if a baby is upset or distressed.

Doctors were concerned that if pain is highly situational it may not be appropriate to include it in a core outcome set.

Researchers did feel that comfort could be measured, but felt that the long term effects have not been conclusively proven.

Vote: In 14%

Out 86%

Result: Not included in core outcome set

Seizures

Delphi results reviewed.

Doctors discussed the difficulties that can be found diagnosing seizures and the discrepancy between clinical and electrical seizures. They mentioned that seizures have multiple different causes, effects and implications.

Researchers identified that seizures can be very difficult to measure. They confirmed that seizures would be included as an adverse event in most trials.

Vote: In 0%

Out 100%

Result: Not included in core outcome set

BORDERLINE OUTCOMES

Suffering

Delphi results reviewed.

Former patients and parents felt that this was a very vague outcome that was very broad.

Nurses and therapists discussed how maintaining comfort was a crucial aspect of care.

Doctors commented that there was no accepted measure for this outcome.

Vote: In 0%

Out 100%

Result: Not included in core outcome set

Parental bonding with their baby

Delphi results reviewed.

Former patients and parents felt that baby-parent relationships are very important, and some measure relating to parental bonding or involvement should be included somewhere in the outcome set as it is such a key aspect of care. They highlighted that abnormal bonding can have lifelong effects.

All groups were concerned that this outcome would not be measurable.

Vote: In 29%

Out 71%

Result: Not included in core outcome set

Resuscitation at birth

Delphi results reviewed.

All groups agreed that this was important, but it was agreed that for most research this would not be an outcome (as it would occur before most research interventions). It was agreed that it was an important background characteristic that should be reported, but that for many trials it would not be an outcome.

Vote: In 0%

Out 100%

Result: Not included in core outcome set

Chronic lung disease or bronchopulmonary dysplasia

Delphi results reviewed.

Former patients and parents discussed that breathing is clearly crucial, but can often be overlooked. Breathing problems can cause life-long issues, even if a baby survives. They highlighted the impact that long term supplemental oxygen can have on a child's life.

Doctors pointed out that this disease can be fatal. They highlighted the absence of any other respiratory outcome from the core outcome set. They discussed that chronic lung disease can be linked to other outcomes (like necrotising enterocolitis and sepsis).

Researchers suggested that this was a very important outcome. They discussed how it correlates to future hospital admissions and later lung function. They accepted that there were major problems with definitions and measures for this outcome, but felt that it was crucial to have a measure for respiratory function in the core outcome set.

Vote: In 100%

Out 0%

Result: Included in core outcome set

Growth

Delphi results reviewed.

Former patients and parents identified that data relating to this outcome was readily available as it is measured continuously during clinical care. However, they did not feel it was their top priority.

Doctors discussed how it was unclear what optimal growth was.

Researchers felt that this was a general measure that did influence other outcomes.

Vote: In 62%

Out 38%

Result: Not included in core outcome set

General communication ability

Delphi results reviewed.

All groups discussed how this outcome did have overlap with other developmental outcomes.

Researchers commented that this outcome was difficult to measure.

Vote: In 8%

Out 92%

Result: Not included in core outcome set

General fine motor ability (writing/dressing etc.)

Delphi results reviewed.

Former patients and parents felt that these were closely linked to developmental milestones.

Nurses and therapists discussed how this outcome was difficult to define.

Doctors commented that these milestones were distinct and separate from the gross motor outcome previously discussed.

Researchers discussed how these milestones could be measured at an early age.

Vote: In 8%

Out 92%

Result: Not included in core outcome set

Normality after discharge

Delphi results reviewed.

Former patients and parents felt that this outcome encompassed a very broad topic. They commented on how “not being normal is not always detrimental”. They were concerned that this outcome could be highly judgemental.

All groups felt that this outcome related to whether individuals were able to participate adequately in society.

Vote: In 0%

Out 100%

Result: Not included in core outcome set

Ability to feed themselves

Delphi results reviewed.

Former patients and parents discussed how this could be a necessity, but they did not feel that it was a priority for research.

Vote: In 8%

Out 92%

Result: Not included in core outcome set

Parental involvement

Delphi results reviewed. Plain English definition reviewed: 'This is whether their parents feel involved in the care of their baby and able to spend as much time with them as they wish'.

Former patients and parents commented that they did not feel this was a core outcome. They did feel that optimising this was an important aspiration for all neonatal care. They highlighted how this can impact on baby-parent bonding.

Researchers discussed how this was more an experience of care than an outcome.

Vote: In 8%

Out 92%

Result: Not included in core outcome set

Tracheostomy

Delphi results reviewed.

Previous discussion about respiratory outcomes reiterated. All groups agreed that this outcome had substantial overlap with 'chronic lung disease/bronchopulmonary dysplasia'.

Vote: In 8%

Out 92%

Result: Not included in core outcome set

Ability to live independently

Delphi results reviewed.

All groups felt that there was significant overlap between this outcome and quality of life. All groups were concerned that measuring this might not be feasible for all research.

Former patients and parents felt that this might be a necessity and could be affected by an individual's context.

Researchers did not feel that this was a better way to measure quality of life.

Vote: In 0%

Out 100%

Result: Not included in core outcome set

Breastfeeding

Delphi results reviewed.

All groups agreed that promoting breastfeeding was very important and that it was something that should be supported throughout neonatal care.

Former patients and parents discussed how breastfeeding could both affect and be affected by a mother's emotional state.

Doctors highlighted that patients and parents had consistently scored breastfeeding as a non-critical outcome.

All groups agreed that while the effects of breastfeeding were very important, it was not necessarily a core outcome in and of itself.

Vote: In 0%

Out 100%

Result: Not included in core outcome set

Need for educational support

Delphi results reviewed.

All groups noted significant overlap with other outcomes (particularly ‘general cognitive ability’).

Vote: In 0%

Out 100%

Result: Not included in core outcome set

Effective communication

Delphi results reviewed. Plain English definition reviewed: ‘This outcome is whether healthcare workers (like nurses and doctors) communicate effectively with parents, former neonatal patients and their wider family.’

Doctors discussed how particularly complex care could impact on effective communication between the healthcare team and parents.

Researchers stated that they felt that this outcome would only be relevant in a small subset of studies.

Vote: In 0%

Out 100%

Result: Not included in core outcome set

Damage due to inadequate blood supply

Delphi results reviewed.

Researchers commented that this outcome should be reported as an adverse event.

All groups felt that this outcome would be contained within the outcome ‘adverse events’.

Vote: In 0%

Out 100%

Result: Not included in core outcome set

Circulatory collapse

Delphi results reviewed.

Researchers commented that they would expect this outcome to always be reported as an adverse event.

All groups felt that this outcome would be contained within the outcome ‘adverse events’.

Vote: In 0%

Out 100%

Result: Not included in core outcome set

Mechanical ventilation

Delphi results reviewed.

Discussion about respiratory outcomes re-summarised.

All groups felt that there was significant overlap with the outcome ‘chronic lung disease/bronchopulmonary dysplasia’.

Vote: In 0%

Out 100%

Result: Not included in core outcome set

Oxygen at discharge home

Delphi results reviewed.

Discussion about respiratory outcomes re-summarised.

Doctors felt that this outcome could be affected by national policies.

Researchers commented that this outcome could be heavily influenced by local service provision.

All groups felt that there was significant overlap with the outcome ‘chronic lung disease/bronchopulmonary dysplasia’.

Vote: In 0%

Out 100%

Result: Not included in core outcome set

Complications of respiratory support

Delphi results reviewed.

Discussion about respiratory outcomes re-summarised.

All groups felt that there was significant overlap with the outcomes ‘adverse events’ and ‘chronic lung disease/bronchopulmonary dysplasia’.

Vote: In 0%

Out 100%

Result: Not included in core outcome set

Other neurological problems

Delphi results reviewed.

All groups felt that this outcome was vague.

All groups felt that there was significant overlap with the outcomes ‘brain injury on imaging’ and the developmental outcomes.

Vote: In 0%

Out 100%

Result: Not included in core outcome set

OUTCOMES IDENTIFIED BY ATTRITION ANALYSIS

Team-working by healthcare professionals

Delphi results reviewed. Attrition analysis results reviewed.

The small number of ‘drop-outs’ was noted. All groups agreed that this reduced the reliability of this scoring pattern. All groups agreed that completing the three rounds of the Delphi surveys allowed for important opportunities for reflection.

No groups felt that this was an important outcome that needed to be included in the core outcome set.

Vote: In 0%

Out 100%

Result: Not included in core outcome set

Effective caring relationships with patients

Delphi results reviewed. Attrition analysis results reviewed. Plain English definition reviewed: ‘this is whether healthcare workers (nurses and doctors) are able to build close relationships with parents.’

The small number of ‘drop-outs’ was noted. The change in scoring patterns over the three rounds was noted (with a general reduction in scores over the three rounds).

No groups felt that this was an important outcome that needed to be included in the core outcome set.

Vote: In 0%

Out 100%

Result: Not included in core outcome set

IV. Final Core Outcome Set

The outcomes that had been included in the core outcome set at this point were reviewed:

Survival

Sepsis

Necrotising enterocolitis

Brain injury on imaging

Retinopathy of prematurity

General gross motor ability (walking/sitting etc.)

General cognitive ability

Adverse events

Visual impairment or blindness

Hearing impairment or deafness

Chronic lung disease or bronchopulmonary dysplasia

These outcomes were reviewed and no significant duplication was identified.

The outcomes were reviewed and compared with the core areas suggested by the OMERACT Filter 2.0. It was agreed that the core areas of 'death', 'pathophysiological manifestations' and 'adverse events' were adequately covered. It was noted that the area of 'resource use/economical impact' is recommended but not strictly core. No stakeholders felt that it was necessary to cover this area in the neonatal core outcome set at this time (although this would be an area to review in the future). All groups noted that the core area of 'life impact' was poorly covered.

Former patients and parents advocated that the outcome quality of life should be reconsidered. They suggested that if it was not included there was no reflection of the "journey taken by patients". Quality of life linked to several highly prioritised outcomes, none of which had come through this consensus process (but were recognised as important nonetheless). Including quality of life would also help to capture some of the complexities of the baby-parent relationship and the impact that could have. Former patients and parents suggested that this outcome could have different measures at different time points, e.g. a short-term measure (on the neonatal unit) could relate to parental involvement in care, and a longer-term measure could be some form of parent-report and/or patient-report questionnaire.

In line with the meeting guidelines one further vote was held considering 'quality of life' in the context of the final core outcome set.

Vote:	In	100%
	Out	0%

Result: Included in core outcome set

No further outcomes were proposed for further consideration.

All outcomes were reviewed in relation to their relevance to babies of different gestational ages. It was unanimously agreed that 'retinopathy of prematurity' and 'chronic lung disease or bronchopulmonary dysplasia' should be measured in all trials involving preterm infants, but would not be relevant in trials only containing term infants. All agreed that they were not advocating that all babies should be screened for retinopathy of prematurity and that local policy should be followed. All other outcomes were relevant to all trials.

The final core outcome set was as follows:

Survival

Sepsis

Necrotising enterocolitis

Brain injury on imaging

Retinopathy of prematurity (preterm only)

General gross motor ability (walking/sitting etc.)

General cognitive ability

Quality of life

Adverse events

Visual impairment or blindness

Hearing impairment or deafness

Chronic lung disease or bronchopulmonary dysplasia (preterm only)

V. Future work

JW then presented the next steps in the COIN project.

The importance of dissemination highlighted. This work to be written up and published as soon as possible. All agreed that we wished to engage with other groups engaged in similar work to try and develop complementary approaches.

JW discussed the issues of outcome measures and time points. Now that the core outcome set has been identified JW will return to the review of outcomes in neonatal clinical trials and assess each outcome measure using the standards in the OMERACT Filter 2.0 (Boers et al., 2014). Measures would be specifically assessed as to whether outcome measures are truthful, discriminative and feasible. The results of this activity will be brought to a further consensus meeting so that an appropriate outcome measure and measurement time point can be recommended for each outcome.

VI. Adjournment

Following these discussions MK sought any further business (of which there was none). The meeting was then adjourned.
